# A dilemma regarding the optimal administration of nimodipine in the subarachnoid hemorrhage

**DOI:** 10.1007/s00701-015-2429-1

**Published:** 2015-05-07

**Authors:** Tomasz Tykocki

**Affiliations:** Department of Neurosurgery, Institute of Psychiatry and Neurology, Sobieskiego Street, Warsaw, 02-957 Poland

Dear Editor,

I have read with interest a recently published article in *Acta Neurochirurgica* by Abboud at al. [1], titled "Serum levels of nimodipine in enteral and parenteral administration in patients with aneurysmal subarachnoid." The authors report a retrospective cohort study of 15 patients, obtaining 157 blood samples. The authors compared serum nimodipine concentrations in patients with aneurysmal subarachnoid hemorrhage (SAH) after parenteral therapy and a following course of enteral administration. Finally, the authors concluded that the area under the curve (AUC) values during parenteral administration (median 149.3 ng-h/ml) were significantly higher than during oral administration on days 9 (median 92.1 ng-h/ml) and 12 (median 44.1 ng-h/ml). They also found that nimodipine AUC values during enteral administration were higher in patients who received nimodine orally than in those who received it by gavage.

Nimodipine is the most widely studied calcium antagonist in SAH, and this original study shed some new light on SAH treatment with the calcium channel blocker nimodipine. Oral nimodipine 60 mg 4 hourly was found to reduce cerebral infarction and improve outcomes after subarachnoid hemorrhage. It was undoubtly proven that oral nimodipine improves the overall outcome [4]. There are no clear data supporting the effectiveness of nimodipine when administered intravenously, and the evidence for other calcium antagonists is inconclusive. Intravenous administration of calcium antagonists cannot be recommended for routine practice on the basis of the present evidence. However, this conclusion might require further investigation, and trials with large patient cohorts would be decisive.

Another calcium antagonist, nicardipine, was associated with a significant and sustained reduction in mean cerebral blood flow velocity as measured by transcranial Doppler when used in the treatment of suspected cerebral vasospasm following aneurysmal subarachnoid hemorrhage [6]. However, in randomized double-blind trials, intravenous nicardipine has been shown to provide less favorable outcomes [2].

The issue of the optimal administration of nimodipine has been debated in the literature, and the results promote oral therapy as the most beneficial. However, patients with higher Hunt-Hess grades (IV or V) are at risk of delayed gastric emptying and gastrointestinal uptake of nimodipine during the first week after subarachnoid hemorrhage. The authors have clearly proven that when considering only the serum nimodipine concentration level and AUC, intravenous administration might be more beneficial. However, in the study by Abboud et al. [1], the authors did not perform a correlation analysis between the nimodipine serum concentration and clinical results including the rate of vasospasm or delayed ischemic neurologic deficits, which makes it difficult to use these data in clinical practice. The authors mentioned that they had not included the clinical evaluation in this comparison because of the small number of patients.

The clinical effect of nimodipine results in a reduction of the rate of vasospasm and associated secondary ischemia. Based on the previous studies, there was no significant difference between the enteral versus intravenous group in the incidence of delayed ischemic neurologic deficits, middle cerebral artery blood flow velocities, number of new ischemic lesions or clinical outcome [3, 5]. The interesting issue regarding the article by Abboud et al. is how these results could be transferred to daily practice in light of the well-known clinical data on the administration of nimodipine. There is also another important question about the discrepancy between the serum nimodipine concentration level and clinical outcome. These probably require new studies for clarification.
